# Modified Tal Score as a Predictor of Outcome in Bronchiolitis: A Cross-Sectional Study in Nepal

**DOI:** 10.7759/cureus.69595

**Published:** 2024-09-17

**Authors:** Madan Kumar Timalsena, Baburam Pandey, Milan Dhungana, Gurbi Khanal, Dinesh Neupane, Bibek Parajuli, Ritika Shrestha

**Affiliations:** 1 Pediatrics, Kist Medical College, Lalitpur, NPL; 2 Pediatrics, Universal College of Medical Sciences, Bhairahawa, NPL; 3 Pediatrics, Chitwan Medical College, Bharatpur, NPL; 4 Internal Medicine, Gandaki Medical College, Pokhara, NPL; 5 Internal Medicine, Universal College of Medical Sciences, Bhairahawa, NPL

**Keywords:** bronchiolitis, escalated care, length of hospital stay, modified tal score, picu admission

## Abstract

Introduction

Bronchiolitis is a leading cause of respiratory distress and hospitalizations among infants and young children worldwide. Despite its prevalence, predicting the severity and outcomes of bronchiolitis remains challenging. Standardized criteria for severity assessment are essential for improving patient care and resource allocation. The modified Tal score is a reliable tool for assessing the severity of bronchiolitis. This study examines the relationship between the modified Tal score at presentation and key clinical outcomes in children with bronchiolitis.

Methods

This cross-sectional study classified children with bronchiolitis into mild, moderate, and severe groups using the modified Tal score at presentation. Patients were followed to record the length of hospital stay, duration of oxygen therapy, and the need for pediatric intensive care unit (PICU) or escalated care.

Results

Of the 121 children analyzed, the majority (71.9%) were aged three to 11 months, with a mean age of 9.09 months, and 73.6% were male. Oxygen supplementation was required by 60.3% of participants, 10.7% needed PICU admission, and 2.5% required escalated care. The mean duration of oxygen therapy was significantly longer in the severe group compared to the mild and moderate groups (p<0.001). Hospital stays also increased with severity (p<0.001). Linear regression showed that each unit increase in the modified Tal score resulted in a 0.746-day rise in hospital stay, a 10.19-hour increase in oxygen requirement, and a 0.35-day increase in PICU stay (all p<0.001). The score accounted for 66.1% of the variability in hospital stay, 63.0% in oxygen requirement, and 53.3% in PICU stay, but only 9.5% in escalated care.

Conclusion

A higher modified Tal score at admission was associated with prolonged hospitalization, increased oxygen requirement duration, and greater need for PICU admission in bronchiolitis. However, the modified Tal score was not a strong determinant of the requirement for escalated care.

## Introduction

Bronchiolitis is a common respiratory illness that primarily affects infants and young children, posing a significant global health burden due to its high morbidity and mortality rates [1]. It is most often caused by viral pathogens, with respiratory syncytial virus (RSV) being the leading cause worldwide, accounting for a substantial number of hospital admissions annually [2]. In Nepal, the situation is equally concerning, with a study conducted in rural areas in 2017 reporting an RSV incidence rate of 213 cases per 1000 person-years among infants [3]. Bronchiolitis is characterized by inflammation of the bronchioles and the small airways in the lungs, leading to airway obstruction and respiratory distress [4-7]. Despite this disease's prevalence and impact, bronchiolitis management remains primarily supportive, with no universally accepted treatment protocols [8]. The complexity of the disease, coupled with the need for objective criteria for assessing severity, further complicates clinical management.

The challenge in managing bronchiolitis lies in accurately assessing disease severity, which is crucial for guiding treatment decisions, predicting outcomes, and optimizing resource allocation in healthcare settings [9]. Several clinical scoring systems, such as the Tal score, modified Tal score (MTS), Lowell score, Wang score, and Liu score, have been developed to address this need [10]. The Tal score, initially developed by Bierman et al. in 1974 to study the pharmacologic management of status asthmaticus in children [11], was further utilized by Asher Tal in 1983 to investigate the effects of dexamethasone and salbutamol in treating acutely wheezing children aged six to twelve months [12]. The initial scoring system included parameters such as respiratory rate, wheezing, cyanosis, and accessory muscle utilization, with scores ranging from zero to three. However, it lacked defined cut-offs for grading severity, and the respiratory rate score was not adjusted for different age groups. In 2012, researchers modified the score by substituting oxygen saturation levels for cyanosis [10]. In 2018, we made further improvements by differentiating the respiratory rate parameter based on age groups, specifically those above and below six months [2]. However, there is ongoing debate regarding the most appropriate and reliable tool for clinical use. Among these, the modified Tal score has garnered attention for its repeatability, internal consistency, and ability to predict clinical outcomes [2,10,13-15]. Previous studies have suggested that the modified Tal score is a robust tool for assessing the severity of bronchiolitis, with studies showing its correlation with oxygen requirement and length of hospital stay.

Despite the promising results of the modified Tal score, there remains a research gap in fully understanding its predictive value in clinical outcomes, such as the duration of oxygen therapy, length of hospital stay, and the need for pediatric intensive care unit (PICU) admission. Current literature provides limited evidence on applying the modified Tal score in predicting these critical outcomes, particularly in low-resource settings where accurate assessment tools are essential for optimizing care.

This study addresses this gap by evaluating the predictive value of the modified Tal score in bronchiolitis outcomes. By analyzing the relationship between the modified Tal score and key clinical outcomes, such as the duration of oxygen requirement, length of hospital stay, and the need for PICU admission, this research seeks to validate the score's utility in guiding clinical management. The findings of this study could have significant implications for improving bronchiolitis care, particularly in resource-limited settings, by providing a reliable, evidence-based tool for clinicians to make informed decisions.

## Materials and methods

Study design

This is a single-centered, cross-sectional, observational study conducted in the emergency room, pediatric ward, and pediatric intensive care unit of KIST Medical College and Teaching Hospital, Nepal, from November 1st, 2021, to March 30th, 2023.

Participant characteristics

Clinically, bronchiolitis is defined as a dry cough, fine inspiratory crackles and/or wheeze, and tachypnea, with or without fever, typically preceded by nasal discharge, in children under 24 months of age. The study included children who presented with a clinical diagnosis of bronchiolitis and required admission at KIST Medical College and Teaching Hospital. Exclusion criteria included children ≤28 days old, those with chronic respiratory conditions such as bronchiectasis, asthma, emphysema, or cystic fibrosis, and those with congenital heart disease or respiratory distress from causes other than bronchiolitis. Children with a history of previous wheezing episodes, families unwilling to provide consent, and those who left the hospital before completing treatment were also excluded from the study.

Ethics approval and consent to participate

This study commenced after ethical approval from the Institutional Review Committee (IRC)-KIST Medical College (Ref. No. 078/079/28). Informed written consent was obtained from parents or guardians before patients were enrolled in the study, and confidentiality of the patient's personal information and the data collected was maintained.

Sample size

The sample size was calculated as:

$n=z^{2\ }\times p\ \times\ \frac{(1-p)}{e^2}$,

Where n is the sample size, z is the confidence interval (95% for this study), p is the prevalence of the condition in a population (taken as 9% based on a previous study done in rural Nepal [3], and e is the margin of error (considering 5% for this study).

The calculated sample size was 126. The total sample collected was 130, of which nine children left treatment early and were excluded from the study. Thus, 121 children were followed up until discharged and analyzed.

Study methods

All patients were managed according to the department's standard protocol. Admission was required for patients presenting with apnea, persistent oxygen saturation below 92% in room air, inadequate oral fluid intake (50-75% of usual volume), or severe respiratory distress (e.g., grunting, marked chest recession, respiratory rate over 70 breaths/minute), as determined by the treating pediatrician. Discharge criteria included clinical stability, adequate oral fluid intake, and maintaining oxygen saturation above 92% in room air for four hours, including during sleep. An investigator recorded demographic data, symptoms, day of illness at admission, examination findings, and the modified Tal score upon presentation using a pre-designed proforma (see Appendix 1, Table 9) (Table 1) [10]. The scoring was performed when the child was calm and not crying before the treatment administration, although treatment was not delayed to document scores. Wheezing was assessed with a Littman’s II stethoscope, respiratory rate was counted for 60 seconds using a stopwatch, SpO_2_ was documented using a pulse oximeter (Choicemmed MD300C53 Pediatric) after observing the child for 60 seconds in room air, and the intercostal retraction was scored by one of the investigators. If SpO_2_ dropped below 90% during assessment, oxygen was administered, and a score of 3 was assigned. The treating pediatrician was blinded to the modified Tal score to avoid bias.

**Table 1:** Modified Tal score. Grading: Mild ≤ 5, moderate 6-10, and severe ≥11.

Score	Respiratory rate		SpO_2_ at room air	Wheeze/fine crepts	Use of accessory muscles
	Age <6 months	Age >6 months			
0	≤40	≤30	≥95	None	None
1	41-55	31-45	92-94	Expiration only	Mild (+)
2	56-70	46-60	90-91	Expiration and inspiration with the stethoscope only	Moderate (++)
3	≥71	≥61	≤89	Expiration and inspiration without the stethoscope	Severe (+++)

After admission, patients were followed up daily, with the investigator assessing the modified Tal score every 24 hours and recording oxygen use, the need for escalated care, or transfer to the PICU. Outcome measures included the duration of oxygen requirement (in hours), length of hospital stay (in days), PICU admission, and the need for escalated care. Escalated care requires any of the following during the hospital stay: high-flow nasal cannula (HFNC), noninvasive ventilation (e.g., continuous positive airway pressure (CPAP) or bilevel positive airway pressure (BiPAP), or mechanical ventilation [16].

Data management and statistical analysis

The pre-designed case proforma collected patient characteristics, clinical and laboratory data, study interventions, and their effects. This data was entered into Microsoft Excel (Microsoft Corporation, Redmond, Washington, USA), checked for errors and completeness, and then transferred to SPSS version 16 (IBM Corp., Armonk, New York, USA) for statistical analysis. The Kolmogorov-Smirnov test assessed the normality of each continuous variable. Outcomes were presented as frequencies, and means were used to compare different groups. The correlation coefficient (r) assessed associations between variables. One-way ANOVA tested significant differences among group averages, and a T-test evaluated statistical significance. Linear regression analysis was used to predict the outcomes (duration of hospital stay, duration of oxygen requirement, duration of PICU requirement, and duration of escalated care requirement) based on the modified Tal score at admission. The regression models included the constant, coefficient, degrees of freedom, F-statistic, adjusted R-squared, and p-values to describe the relationships between the variables. A p-value of <0.05 was considered statistically significant.

## Results

One hundred thirty children who fulfilled the inclusion criteria were enrolled, of which nine children who left treatment early were excluded from the study, so 121 children were followed up till discharge. Most children were three to 11 months, with a mean age of 9.09. Males comprised 73.6% of the participants. The mean duration of illness at admission was 4.54 days (Table 2).

**Table 2:** Age, sex distribution, and mean duration of illness of participants.

Parameter	Value
Mean age (±SD)	9.09 months (±5.38)
Median age (IQR)	9.00 months (5,11)
Age category (months)	
1 m-2 m	5 (4.1%)
3 m-11 m	87 (71.9%)
12 m-23 m	29 (24 %)
Sex	
Male	89 (73.6%)
Female	32 (26.4%)
Mean duration of illness at admission	4.54 days (±2.22)

Clinically, cough was the most common presenting complaint, followed by nasal discharge and fever, whereas tachypnea was the most common finding, followed by chest indrawing and hypoxemia (Table 3).

**Table 3:** Clinical features of study participants.

Symptoms	Frequency (percentage)
Cough	117 (96.7%)
Nasal discharge	90 (74.4%)
Fever	72 (59.5%)
Noisy breathing	71 (58.7%)
Fast breathing	32 (26.4)
Vomiting	5 (4.1%)
Loose stool	4 (3.33%)
Decreased feeding	3 (2.47%)
Ear ache	1 (0.8%)
Lethargy	1 (0.8%)
Chest indrawing	1 (0.8%)
Change in voice	1 (0.8%)
Clinical findings	Frequency (percentage)
Tachypnea	68 (56.2%)
Chest indrawing	42 (34.7%)
Hypoxemia	22 (18.2%)
Tachycardia	13 (10.7%)
Lethargy	4 (3.3%)
Cyanosis	0

The mean modified Tal score at the time of presentation was 4.8. Most participants were in the mild group, followed by the moderate and severe groups (Table 4).

**Table 4:** Baseline characteristics of study participants.

Clinical variables	Values
Mean M Tal score at presentation	4.8 days (±2.5)
Mild (M Tal score ≤5)	84 (69.4%)
Moderate (M Tal score 6-10)	27 (22.3%)
Severe (M Tal score ≥11)	10 (8.3%)
Mean respiratory rate	
1-2 months	60/minute (±13.26)
3-11 months	52.7/minute (±10.5)
12-23 months	47.5/minute (±11.7)
Mean heart rate	
<12 months	140.2/minute (± 20.48)
12-23 months	138.1/minute (±19.5)

Study outcomes

About 60.3% of the participants required oxygen supplementation, 10.7% required PICU admission, and 2.5% required escalated care (Table 5).

**Table 5:** Requirement of oxygen, PICU, and escalated care among different severity groups. PICU: pediatric intensive care unit.

Parameter	Mild (n = 84)	Moderate (n = 27)	Severe (n = 10)	Total (n = 121)	p-value (95%CI)	Chi-square (χ²)
Cases requiring oxygen therapy						
Yes	37 (44%)	26 (96.3%)	10 (100%)	73 (60.3%)	<0.001	27.456
No	47 (56%)	1 (3.7%)	0	48 (39.7%)		
Cases requiring PICU admission						
Yes	0	3 (11.1%)	10 (100%)	13 (10.7%)	<0.001	98.152
No	84 (100%)	24 (88.9%)	0	108 (89.3%)		
Cases requiring escalated care						
Yes	0	1 (3.7%)	2 (20%)	3 (2.5%)	<0.001	15.320
No	84 (100%)	26 (96.3%)	8 (80%)	118 (97.5%)		

The study found that as the severity of the condition increased, so did the mean oxygen requirement, hospital stay, PICU stay, and escalated care, all with p-values <0.001. Severe cases had the highest mean oxygen requirement (81.50 hours) and longest hospital stay (8.80 days). PICU admission was needed only in moderate and severe cases, with severe cases requiring an average of 3.8 days. Escalated care was significant only in severe cases (15.7 hours) (Table 6).

**Table 6:** Mean outcome of the study population. PICU: pediatric intensive care unit.

Parameter	Mild	Moderate	Severe	Total	F	p-value
Mean oxygen requirement (hours) (SD)	11.16 (±16.54)	49.65 (±30.38)	81.50 (±37.06)	25.56 (±32.07)	64.279	<0.001
Mean hospital stay (days) (SD)	3 (±1.16)	5.74 (±1.78)	8.80 (±2.15)	4.09 (±2.29)	98.476	<0.001
Mean PICU requirement (days) (SD)	0	0.26 (±0.86)	3.8 (±1.62)	0.37 (±1.2)	178.550	<0.001
Mean escalated care requirement (hours) (SD)	0	0.7 (±3.6)	15.7 (±35.37)	1.45 (±10.7)	11.292	<0.001

Predicted hospital stay, oxygen requirement, PICU requirement, and escalated care

Regression analyses showed that each unit increase in the modified Tal score (MTS) was significantly associated with increased hospital stay, oxygen requirement, PICU requirement, and escalated care. Specifically, MTS was linked to a 0.746-day increase in hospital stay (R² = 0.661), a 10.19-hour increase in oxygen requirement (R² = 0.63), a 0.35-day increase in PICU requirement (R² = 0.533), and a 1.373-hour increase in escalated care. The associations between hospital stay, oxygen requirement, and PICU requirement were strong and statistically significant (p < 0.001), but the relationship with escalated care was weaker, explaining only 9.5% of its variability (adjusted R² = 0.095) (Table 7).

**Table 7:** Predicted hospital stay, oxygen requirement, PICU requirement, and escalated care requirement of the study population using linear regression. PICU: pediatric intensive care unit.

Parameter	Constant (±SD)	Coefficient (±SD)	F	Adjusted R square (R²)	p-value (95% CI)
Hospital stay (days) SD	0.507 (±0.26)	0.746 (±0.49)	235	0.661	<0.001
Duration of oxygen requirement (hours) SD	−23.408 (±3.839)	10.199 (±0.709)	206.63	0.63	<0.001
PICU requirement (days) SD	−1.31 (±0.16)	0.35 (±0.30)	137.92	0.533	<0.001
Escalated care requirement (hours) SD	−5.136 (±2.23)	1.373 (±1.46)	13.60	0.095	<0.001

Table 8 shows the predicted duration of hospital stay (days), duration of O_2_ requirement (hours), PICU stay (days), and duration of escalated care (hours) in the general population.

**Table 8:** Predicted duration of hospital stay (days), duration of O2 requirement (hours), PICU stay (days), and duration of escalated care (hours) in the general population. PICU: pediatric intensive care unit. Predicted regression model was y = a + bx, x = modified Tal score at admission. For the predicted duration of hospital stay (y), a = 0.507, b = 0.746. For the predicted duration of oxygen requirement (y), a = −23.408, b = 10.199. For the predicted PICU stay (y), a = −1.31, b = 0.35. For the predicted duration of escalated care (y), a = −5.136, b = 1.373.

Modified Tal score	Predicted duration of hospital stay (days)	Predicted duration of O_2 _requirement (hours)	Predicted PICU stay (days)	Predicted duration of escalated care (hours)
1	1.25	−13.20	−0.96	−3.76
2	2	−3	−0.61	−2.39
3	2.74	7.18	−0.26	−1.01
4	3.49	17.38	0.09	0.35
5	4.23	27.58	0.44	1.72
6	4.98	37.78	0.79	3.10
7	5.72	47.98	1.14	4.47
8	6.47	58.18	1.49	5.84
9	7.22	68.38	1.84	7.22
10	7.96	78.58	2.19	8.59
11	8.71	88.78	2.54	9.96
12	9.45	98.98	2.89	11.34

## Discussion

This study provides insights into the clinical profile and outcomes of bronchiolitis in a cohort of 121 children, focusing on the utility of the modified Tal score in predicting the severity and duration of illness, oxygen requirements, and the need for intensive care.

Our findings regarding the mean age and male predominance are consistent with other studies that have reported a mean age of around ten months and a higher incidence in males [14,17]. Other studies have also reported lower mean ages, ranging from four to five months, and male preponderance [2,10,18]. This suggests that younger infants may be at higher risk for severe viral respiratory infections, and the male incidence observed across various studies has been attributed to biological factors, such as higher viral loads and less robust immune responses in males compared to females [19].

The duration of illness and predominant presenting features like cough, nasal discharge, and fever in our study are comparable to those reported in the literature [10,20,21]. A notable discrepancy exists between parental recognition of symptoms and clinical findings. While fast breathing and chest indrawing were observed in many children during clinical examination, parents underreported these symptoms [20-22]. This highlights a gap in parental awareness and the potential role of healthcare education in improving early recognition of severe respiratory illness [22].

The distribution of bronchiolitis severity in our cohort, predominantly in the mild category, aligns with previous studies [18]. The relationship between the modified Tal score and oxygen requirement was significant, with higher scores correlating with longer oxygen therapy durations. These findings are consistent with other studies showing a progressive increase in oxygen needs with the severity of bronchiolitis [2,18,23]. The variability in oxygen requirement duration across different studies may be influenced by differences in healthcare practices, including the timing of intervention and criteria for oxygen supplementation.

Our study's overall mean duration of hospital stay was comparable to previous findings [18], although variations were observed when analyzed by severity [2,17]. The association between higher modified Tal scores and more extended hospital stays reinforces the score's utility in clinical decision-making. This is further supported by regression analysis, which demonstrated a significant relationship between the Tal score and length of stay [23].

Regarding the need for PICU admission, our findings of a 10.7% admission rate are similar to those reported in other studies [20]. However, the variability in PICU stay duration and the relatively low R-squared value suggests that factors beyond the modified Tal score, such as underlying comorbidities and early interventions, might influence the need for intensive care.

The study also examined the role of the modified Tal score in predicting the need for escalated care, such as bubble CPAP or HFNC therapy [9]. Although the score was associated with higher care requirements in severe cases, the low R-squared value indicated that it might not be the sole predictor. Previous studies have identified additional risk factors, including low oxygen saturation, apnea, and prematurity, that are crucial in determining the need for more intensive interventions [16,18].

The strengths of this study include its prospective design, substantial sample size, and the use of linear regression analysis to evaluate the modified Tal score's predictive validity for key clinical outcomes in bronchiolitis, particularly in resource-limited settings. Notably, the study explored the relationship between the modified Tal score at admission and the need for PICU admission and escalated care, which has not been previously studied.

However, the study faced limitations, including a reduced sample size due to the COVID-19 pandemic and consecutive non-probability sampling. Additionally, the predominance of mild cases may have influenced the findings related to PICU and escalated care requirements.

The study suggests that while the modified Tal score is a valuable prognostic tool for bronchiolitis outcomes, clinical judgment and consideration of other factors remain essential. Future research with larger multicenter cohorts and additional variables would enhance the score's predictive utility in clinical practice.

## Conclusions

This study demonstrates a significant relationship between the modified Tal score at admission and clinical outcomes in children with bronchiolitis. A higher score was associated with longer hospitalization, increased oxygen supplementation, and extended PICU stays. The predictive model based on the modified Tal score effectively forecasts hospital stay and oxygen needs, although its ability to predict escalated care is limited. These findings highlight the modified Tal score as a valuable tool for early risk stratification, enabling better resource planning and improved patient outcomes in clinical settings.

## Appendices

 Appendix 1

**Table 9:** Pre-designed pro-forma. PICU: pediatric intensive care unit.

Patient details								
S. No:								
IP No.:								
Date (y/m/d):								
Name:								
Age/sex:								
Address:								
Symptoms				Duration				
Nasal discharge (yes/no)				In days				
Cough (yes/no)				In days				
Noisy breathing (yes/no)				In days				
Fever (yes/no)				In days				
Cyanosis (yes/no)				In days				
Apnea (yes/no)				In days				
Others (specify):				In days				
Day of illness at admission:								
Indication of admission:								
Examination findings								
Pulse:				In beats/min				
Pallor:				(Present/absent)				
Edema:				(Present/absent)				
Dehydration:				(No/some/severe)				
Cyanosis:				(Present/absent)				
Heart sounds and murmur:				(Present/absent)				
Hepatomegaly:				(Present/absent)				
Weight:				In Kg				
Height:				In Cm				
Others (specify):								
Modified Tal score:								
Score assigned	Respiratory rate		Spo_2_ (at room air)		Wheeze/fine crepts	Accessory muscle use		Total score
	Age < 6 M	Age > 6 M						
0	≤40	≤30	≥95		None	None		
1	41-55	31-45	92-94		Expiration only	Mild (+)		
2	56-70	46-60	90-91		Expiration and inspiration with stethoscope only	Moderate (++)		
3	≥71	≥61	≤89		Expiration and inspiration without stethoscope	Severe (+++)		
At admission								
1st/2nd/3rd DOA								
Oxygen requirement: yes/no (if yes)				Maximum flow rate: in lit/min				
O_2_ started at				(Date and time)				
O_2_ stopped at				(Date and time)				
Total hours on oxygen				In hours				
Need of PICU admission				Yes/no (if yes)				
Transferred to PICU at				(Date and time)				
Transferred out of PICU at				(Date and time)				
Total days/hours at PICU								
Need of escalated care				Yes/no (if yes)				
Started at				(Date and time)				
Stopped at				(Date and time)				
Total duration				In hours				
Treatment given				Duration			Indication	
Oral/IV antibiotics								
Nebulization								
IV fluids								
Others								
Date of discharge:								
Total days at hospital:								
Condition at discharge/type of discharge:								
Documented by (name and designation):								
